# Bioinformatics analysis reveals shared gene signatures and molecular mechanisms between periodontitis and rheumatoid arthritis in the context of aging

**DOI:** 10.2340/aos.v85.46622

**Published:** 2026-08-21

**Authors:** Hanlu Xiao, Yajun Zhao, Yishu Wang, Yuhan Wang, Dingxin Zhang, Rongzeng Huang, Chengwu Song, Shan Cao, Shuna Jin

**Affiliations:** a School of Pharmacy, Hubei University of Chinese Medicine, Wuhan, China; b Department of Stomatology, Hubei Provincial Hospital of Traditional Chinese Medicine, Wuhan, China; c Hubei Shizhen Laboratory, Wuhan, China; d School of Traditional Chinese Medicine, Hubei University of Chinese Medicine, Wuhan, China; e School of Basic Medical Sciences, Hubei University of Chinese Medicine, Wuhan, China

**Keywords:** periodontitis, rheumatoid arthritis, aging, bioinformatics analysis, immune infiltration

## Abstract

**Objective:**

The interplay between periodontitis (PD) and rheumatoid arthritis (RA) is well recognized, with aging being a central risk factor for both. However, the shared aging-related molecular mechanisms remain unclear. We aimed to identify common aging-related hub genes linking PD and RA and elucidate their underlying functional mechanisms.

**Material and methods:**

PD and RA gene expression profiles were obtained from Gene Expression Omnibus database. Crosstalk genes were identified via differential expression analysis and intersected with aging-related genes from GeneCards. Functional enrichment (GO/KEGG) was performed. Hub genes were screened using protein–protein interaction network analysis and two machine learning algorithms and validated with external datasets. Diagnostic performance was evaluated by nomogram and receiver operating characteristic analysis. Immune cell infiltration was analyzed using CIBERSORT.

**Results:**

We identified 191 aging-related crosstalk genes, enriched in immune-inflammatory pathways. Five hub genes (RAC2, CSF2RB, IL2RG, ENTPD1, and IL10RA) were confirmed, showing significant overexpression in PD and RA tissues with excellent diagnostic value. Immune infiltration revealed coordinated dysregulation of plasma cells and γδ T cells in both diseases.

**Conclusions:**

This study reveals shared aging-related genes, pathways, and immune cell dysregulation between PD and RA, elucidating how aging exacerbates both diseases through immune-inflammatory mechanisms. These findings provide a theoretical basis for novel biomarkers and therapies.

## Introduction

Periodontitis (PD), a chronic inflammatory condition triggered by microbial plaque biofilm, leads to the breakdown of periodontal tissues. Its hallmark features encompass alveolar bone resorption, the development of periodontal pockets, progressive attachment loss, tooth mobility, and eventual tooth loss [1]. PD ranks as the sixth most prevalent disease globally, affecting 20–50% of the population [2], with its severe form impacting approximately 11% [3]. It correlates with several chronic inflammatory diseases, such as cardiometabolic diseases, neurodegenerative disorders, autoimmune diseases, and cancer [4]. Rheumatoid arthritis (RA) is a systemic inflammatory autoimmune disease, pathologically characterized by synovial inflammation and joint destruction and clinically associated with high autoantibody titers [5]. With a global prevalence around 0.46% [6], RA commonly leads to serious complications involving cardiovascular, skeletal, and pulmonary diseases [7].

Recent studies have revealed a significant interaction between PD and RA. On the one hand, PD has been established as a risk factor that contributes to the development and exacerbation of RA [8]. Compared to healthy controls, patients with PD exhibit approximately a 1.09–1.69-fold higher risk of being diagnosed with RA [9–11], and their RA disease activity scores are remarkably elevated [12]. On the other hand, RA can also aggravate periodontal lesions; 63.6–75% of RA patients suffer from moderate to severe PD [13–15], and the degree of periodontal destruction is more pronounced in patients with severe RA [16]. Furthermore, the two diseases share similar pathophysiological mechanisms: dysregulation of chronic immune inflammation leads to increased infiltration of neutrophils, macrophages, and T and B lymphocytes [8]. This promotes the production of pro-inflammatory cytokines and stimulates bone-resorbing activity, ultimately resulting in the destruction of the soft and hard tissues of the alveolar bone in PD and the joints in RA [17]. The key pathogen *Porphyromonas gingivalis* not only induces periodontal dysbiosis but may also participate in RA pathogenesis, particularly in anti-citrullinated protein antibody (ACPA)-positive RA, by promoting protein citrullination [18, 19].

It is noteworthy that both PD and RA exhibit significant age-dependent patterns. Aging (or senescence), characterized by progressive deterioration in organismal structure and function [20], may amplify the mechanisms described above, such as PD-RA immune-inflammatory and bone destruction axes, thereby contributing to their comorbidities. Epidemiologically, the prevalence of PD increases significantly in the 35–44 age group, while the increasing trend tends to stabilize in individuals ≥ 60 years old [21]. Nevertheless, the severity of periodontal bone destruction continues to worsen with age [22], possibly because aging-related immune dysregulation promotes osteoclast activation [23, 24]. The incidence of RA also rises significantly in individuals over 50 years old [25]. Moreover, RA patients exhibit features of accelerated aging, with cellular aging estimated to be approximately 20 years ahead [26], and senescent T cells are more prone to drive the progression of synovial inflammation in RA [27]. Collectively, these findings suggest that aging and the accompanying immune-inflammatory changes may represent a key mechanism underlying the co-occurrence of PD and RA and amplify the mutual risk and severity of these two diseases.

However, most current studies still focus on a single disease or a specific molecular pathway, failing to systematically explain how aging integrates and amplifies the common immune inflammation and tissue destruction mechanisms shared by PD and RA and thereby affects the progression of both diseases. With the growth and aging of the population, the disease burden of PD and RA will continue to increase [28, 29]. It is particularly necessary to deeply elucidate the common pathogenic basis of these two diseases in the context of aging. Based on the above reasons, this study comprehensively identified shared hub genes with aging-related expression signatures in PD and RA using bioinformatics techniques and machine learning methods. The potential comorbidity mechanisms underlying the relationship between these two diseases and the aging process were further elucidated. The research results were verified in multiple dimensions using external datasets and receiver operating characteristic (ROC) curve analysis, laying the foundation for subsequent related studies.

## Materials and methods

### Data acquisition and preprocessing

**Figure 1:**
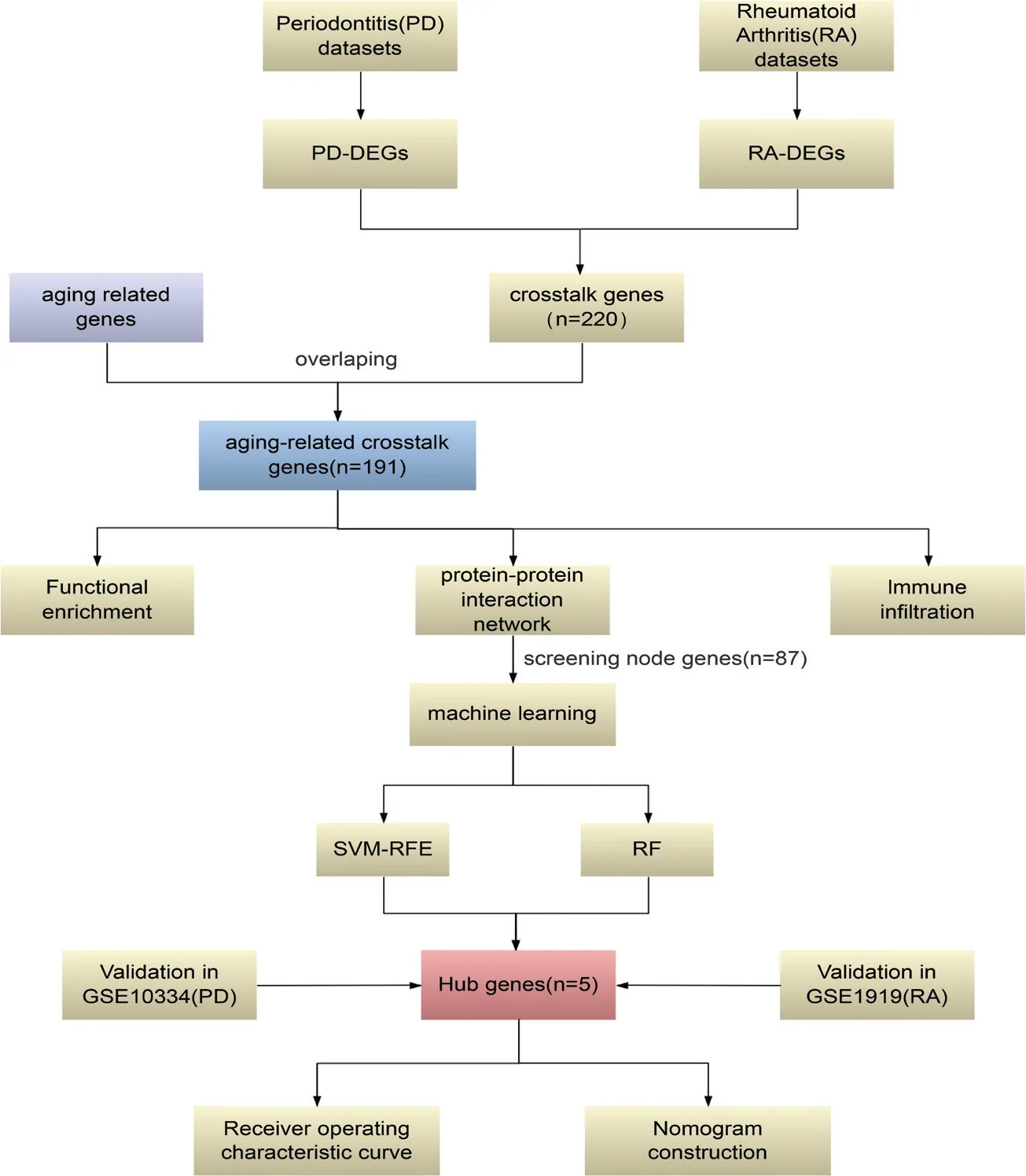
The work flowchart of the study. PD-DEGs, differentially expressed genes in periodontitis; RA-DEGs, differentially expressed genes in rheumatoid arthritis; aging-related crosstalk genes, the intersection between PD-RA crosstalk genes and aging-related genes curated from the GeneCards database. SVM-RFE: support vector machine-recursive feature elimination; RF: random forest; GSE: gene expression omnibus series. PD-DEG: periodontitis-differentially expressed genes; RA-DEG: rheumatoid arthritis-differentially expressed genes; PD-RA: periodontitis-rheumatoid arthritis.

The workflow of this research was outlined in Figure 1. Datasets related to gene expression for both PD and RA were obtained from the Gene Expression Omnibus (GEO) database (https://www.ncbi.nlm.nih.gov/geo/). We used these keywords to search for PD datasets: ‘periodontitis’, ‘human genome’, and ‘gingival tissue’. The datasets were screened according to the following standards: (1) samples originated from *Homo sapiens*; (2) raw data or matrix files were available from GEO, using microarray as the experimental platform; and (3) datasets contained both healthy control and diseased individuals. The search for RA datasets made use of these keywords: ‘rheumatoid arthritis’, ‘human genome’, and ‘synovial tissue’, applying the same filtering standards as for PD. Shared genes were extracted from the RA datasets GSE55235, GSE55457, GSE12021, and GSE77298, and the batch effect of the consolidated data was adjusted using the ComBat algorithm in the sva package (R software, version 4.4.3) to minimize technical variations across the different sample sources.

Supplementary Table 1 detailed each GEO dataset. GSE10334 and GSE1919 were designated as validation datasets for PD and RA to validate the ultimately identified hub genes, respectively.

The GeneCards database (https://www.genecards.org/) serves as a comprehensive and searchable bioinformatics platform. It aggregates information from various repositories to offer detailed, accessible data on both known and predicted human genes [30]. Based on the GeneCards database and using a screening threshold of relevance score ≥ median value, a total of 17,531 aging-related genes were collected for this study (Supplementary Table 2).

### Differential expression analysis

Differential expression screening was conducted using limma, a method grounded in a generalized linear modeling framework [31]. The batch-adjusted datasets for PD and RA were analyzed with the limma R package (version 3.40.6) for this purpose. We identified differentially expressed genes (DEGs) in the PD and RA datasets with a threshold of *p* < 0.05 and |log_2_(FC)| > 0.6. Volcano plots and heatmaps were generated using the ‘ggplot2’ and ‘pheatmap’ packages, respectively.

### Functional enrichment analysis

Functional enrichment analysis of gene sets was performed using Gene Ontology (GO) annotations, sourced from the org.Hs.eg.db R package (version 3.1.0), and current Kyoto Encyclopedia of Genes and Genomes (KEGG) pathway annotations, which were retrieved through the KEGG Representational State Transfer (REST) Application Programming Interface (API) (https://www.kegg.jp/kegg/rest/keggapi.html). Genes were then aligned against this combined background, and a systematic enrichment analysis was conducted using the clusterProfiler package in R (version 3.14.3) to derive the gene set enrichment outcomes. Statistical significance thresholds were set as follows: a minimum of 5 and a maximum of 5000 genes per set, with statistical significance defined by a *p*-value below 0.05 and an FDR (false discovery rate) under 0.25.

### Construction of protein–protein interaction networks

Aging-related crosstalk genes were uploaded to the STRING database (http://www.string-db.org/) for building a protein–protein interaction (PPI) network, depicting both direct and indirect interaction relationships among proteins [32]. A minimum required interaction score≥0.4 was set as the threshold for network construction. The network was visualized using Cytoscape software (version 3.9.1). The top 100 nodes (genes) were scored using five algorithms implemented via the Cytoscape plugin CytoHubba: Maximal Clique Centrality (MCC), maximum neighborhood component (MNC), density of maximum neighborhood component (DMNC), degree, and edge percolated component (EPC). Intersecting candidate genes derived from these five algorithms were selected with the ‘UpSet’ in R and used for subsequent analysis [33].

### Machine learning

We utilized two machine learning approaches to further filter out potential key genes involved in both crosstalk and aging. Support vector machine-recursive feature elimination (SVM-RFE) serves as an effective feature selection algorithm, assigning scores to individual features by leveraging SVM’s maximum margin principle and eliminating redundant ones [34]. Random Forest (RF) is an ensemble learning algorithm made up of numerous decision trees. It is not limited by variable distributions or related assumptions, shows strong performance in terms of accuracy, specificity, and sensitivity; works for continuous variable prediction; and delivers highly reliable prediction outcomes [35].

The R packages ‘e1071’, ‘caret’ and ‘kernlab’ were used for the SVM-RFE algorithm [36]; ‘RandomForest’ package was used for the RF algorithm [37]. Based on the PD and RA datasets, Venn diagram analysis was used to find the intersections of results from each algorithm. The common intersecting genes for both diseases were ultimately identified as the hub genes for PD and RA.

### Expression levels and diagnostic value of candidate biomarkers

We used the ‘ggplot2’ package within R software to examine the expression levels of the identified hub genes in PD and RA samples. A nomogram was constructed with the ‘rms’ package, where ‘Points’ represent individual scores for each candidate gene, and ‘Total Points’ denote the cumulative sum of the scores assigned to these genes.

To evaluate the diagnostic efficacy of potential biomarkers in the PD and RA datasets, we generated ROC curves and calculated the area under the curve (AUC) by means of the ‘pROC’ package. Furthermore, two external datasets (GSE10334 and GSE1919) were used to verify the diagnostic efficiency of the potential biomarkers.

### Immune cell infiltration analysis

CIBERSORT is a computational approach for deconvoluting the proportions of immune cell subsets within a tissue based on its gene expression profile, used here to determine immune cell proportions in disease versus normal groups [38]. We conducted immune cell infiltration analysis with the help of the ‘Cibersort’ package in R software. Bar plots were used to visualize the proportion of each immune cell type across different samples. Box plots were used to visualize comparisons of proportions of different immune cell types between disease and normal groups. We selected infiltrating immune cells with a *p*-value < 0.05 and conducted Spearman correlation analysis to evaluate how these cells correlate with the hub genes.

### Statistical analysis and visualization

Data analysis and visualization were carried out with the use of RStudio (version 4.4.3), SPSS (version 27.0.1), and Sangerbox (http://vip.sangerbox.com/home.html). Spearman correlation analysis was applied. All data were expressed as mean ± standard deviation, with a *p*-value < 0.05 regarded as statistically significant.

## Results

### Data preprocessing

**Figure 2:**
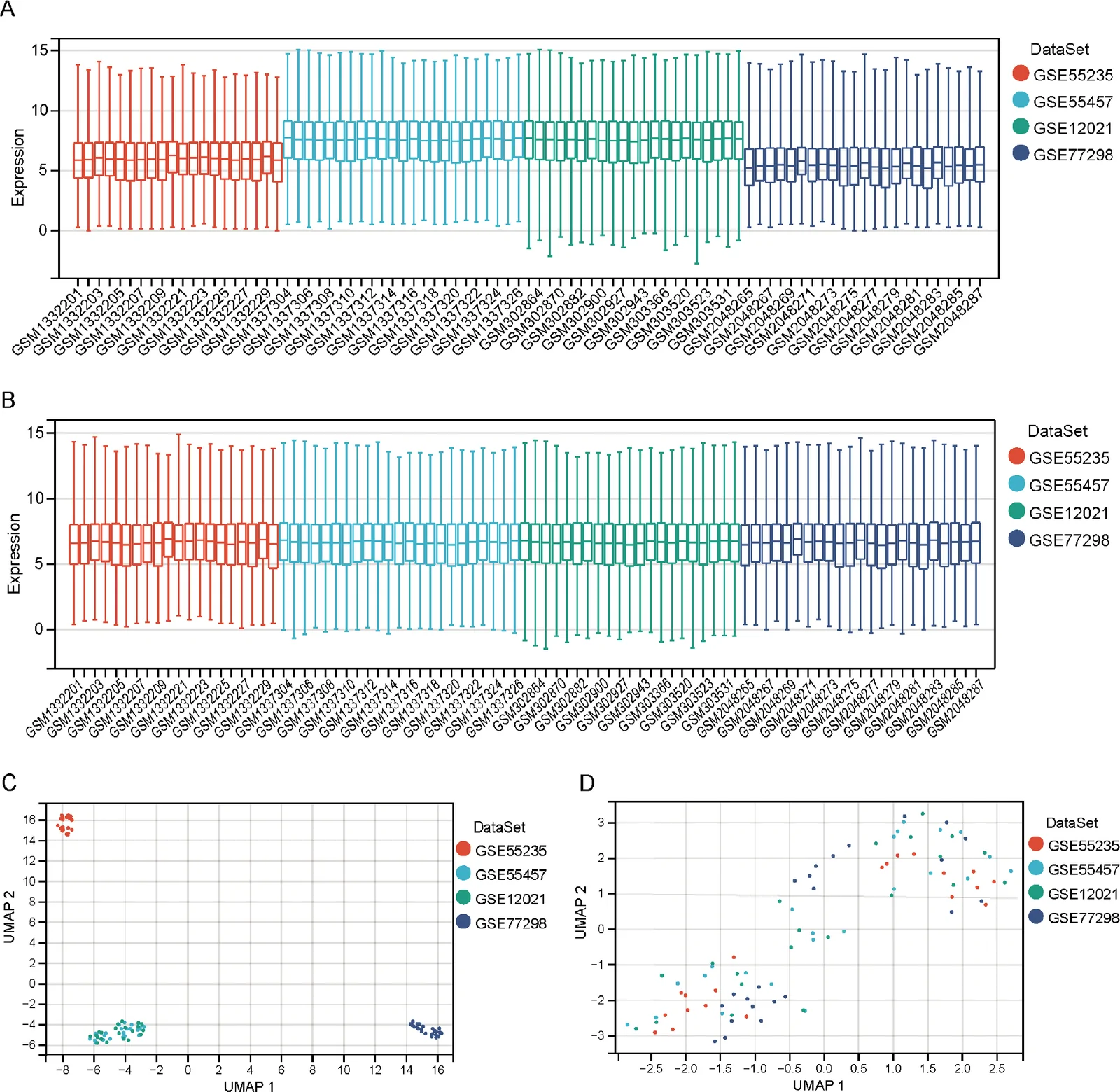
Data preprocessing. (A) The boxplot before removing the batch effect; (B) the boxplot after removing the batch effect; (C) the UMAP plot before removing the batch effect; and (D) the UMAP plot after removing the batch effect.

The PD dataset consisted of GSE16134, which included 241 patient samples and 69 normal control samples in total. The RA datasets included GSE55235, GSE55457, GSE12021, and GSE77298, encompassing a total of 51 patient samples and 36 normal control samples. After applying the ComBat method to reduce batch effects, the boxplots of the RA datasets exhibited greater uniformity in data distribution across the different datasets, with their medians aligned along a straight line (Figure 2A and B). In the Uniform Manifold Approximation and Projection (UMAP) plots, samples from different datasets intermingled, and the discrepancies among the RA datasets were notably diminished (Figure 2C and D).

### Identification of DEGs

**Figure 3:**
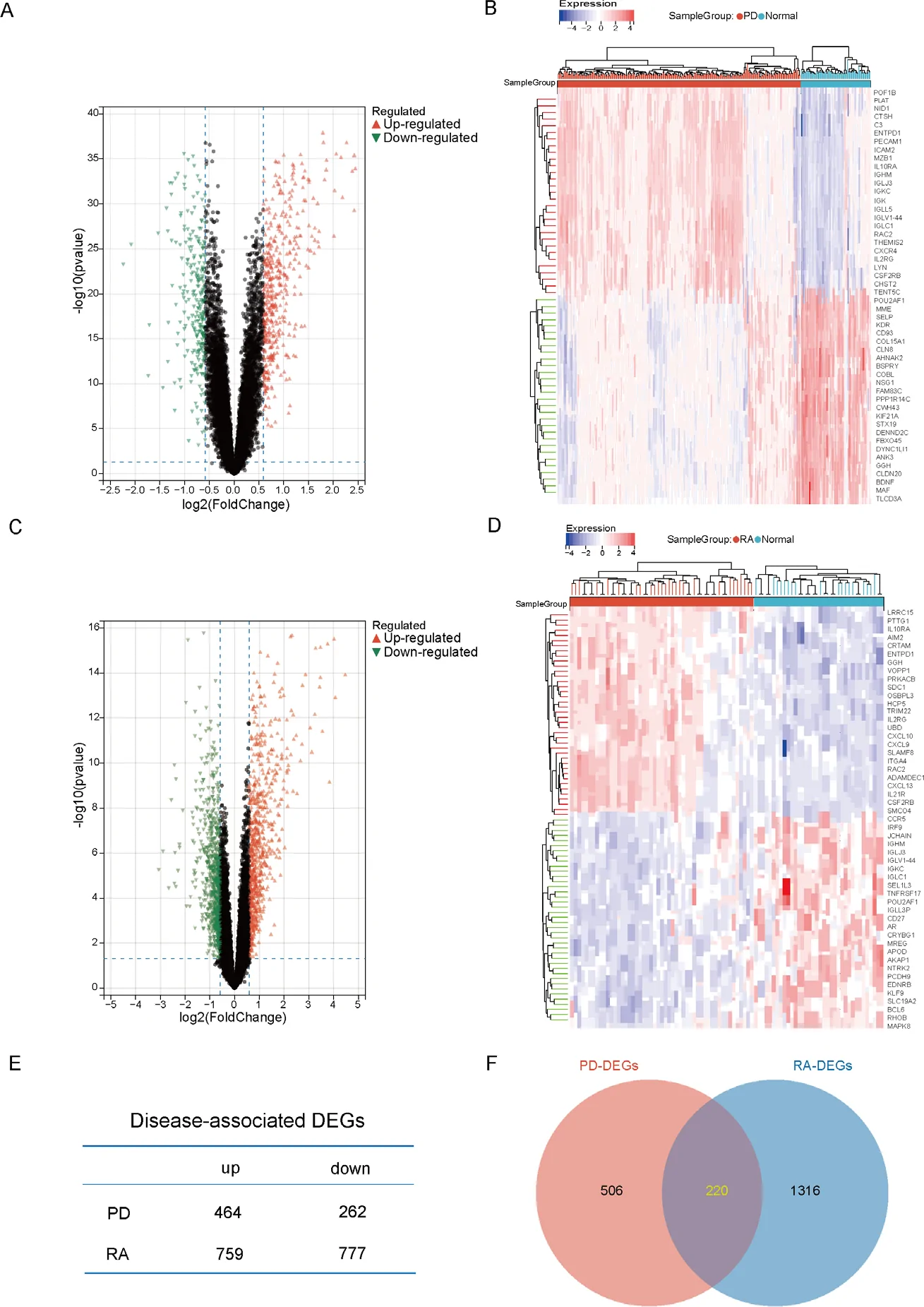
Identification of DEGs. (A) A volcano plot of all DEGs in PD. (B) The heatmap plot of all DEGs in PD. (C) A volcano plot of all DEGs in RA. (D) The heatmap plot of all DEGs in RA. (E) Numbers of upregulated and downregulated disease-associated DEGs identified in PD and RA compared with healthy controls. (F) The Venn diagram of DEG intersection between PD and RA.

Through differential expression analysis, we identified 726 DEGs in PD, comprising 464 upregulated genes and 262 downregulated genes. In RA, 1536 DEGs were obtained, consisting of 759 upregulated genes and 777 downregulated genes (Figure 3E). Volcano plots and heatmaps were utilized to illustrate the expression profiles of the DEGs in both diseases (Figure 3A–D). A Venn diagram revealed 220 shared DEGs between PD and RA, designated as crosstalk genes (Figure 3F).

### Identification of aging-related crosstalk genes and functional enrichment

**Figure 4:**
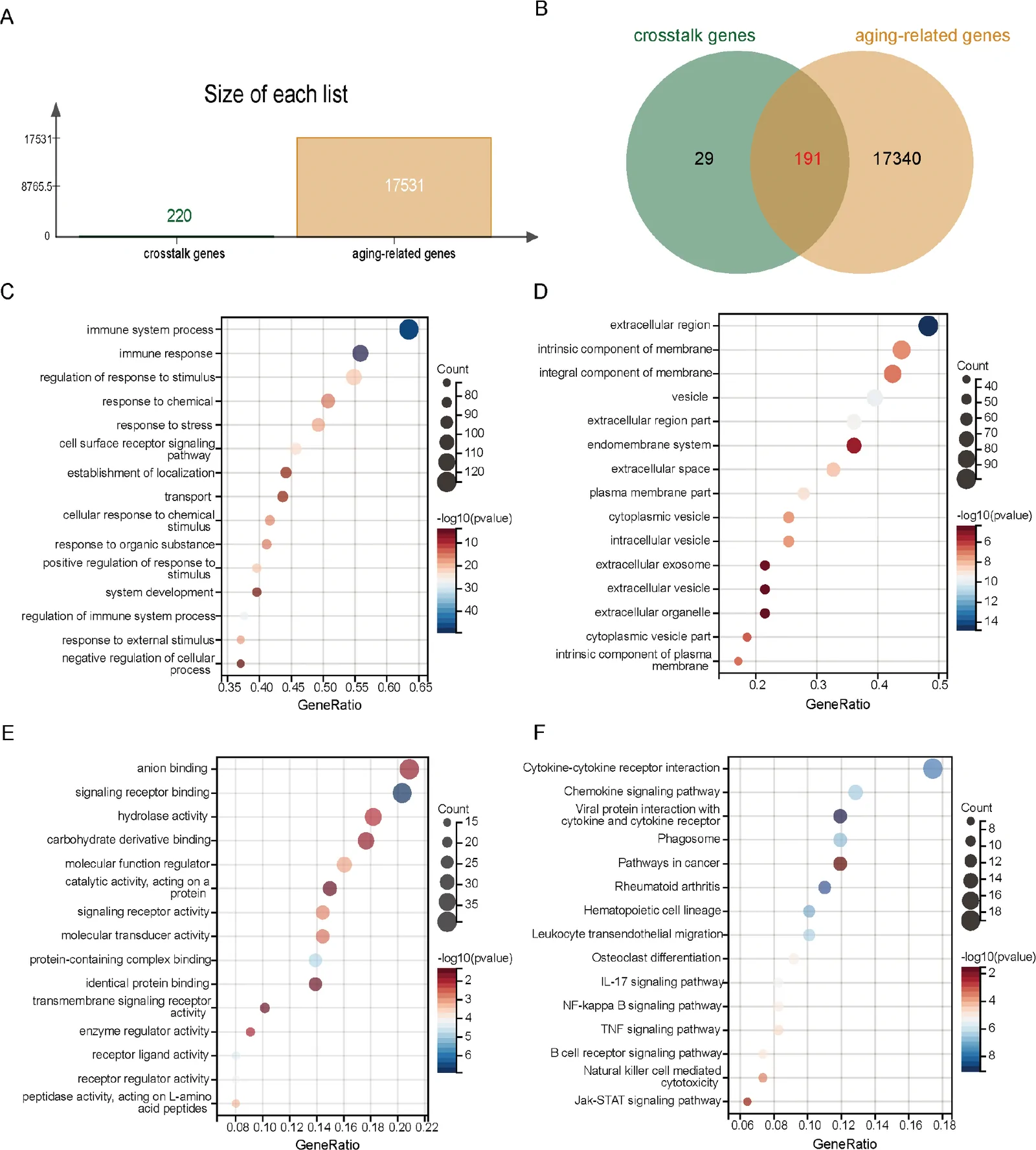
Identification and functional enrichment of aging-related crosstalk genes. (A) Number of genes at the intersection of crosstalk genes and aging-related genes. (B) Venn diagram at the intersection of crosstalk genes and aging-related genes. (C–E) GO analysis results of aging-related crosstalk genes, including top 15 BP terms, top 15 CC terms and top 15 MF terms. (F) The top 15 KEGG pathways of aging-related crosstalk genes were represented. GO: Gene Ontology; BP: biological process; CC: cellular components; MF: molecular function.

Intersecting the crosstalk genes with 17,531 aging-related genes obtained from GeneCards yielded 191 aging-related crosstalk genes (Figure 4A and B). GO and KEGG pathway enrichment analyses were employed to investigate the biological properties and involved pathways of these genes. The top 15 terms of each category were displayed. GO enrichment analysis demonstrated that in terms of biological processes (BP), these aging-related crosstalk genes were mainly concentrated in immune system processes, immune response, regulation of response to stimulus, and response to chemical (Figure 4C). For cellular components (CC), significant enrichment was observed for extracellular region, intrinsic component of membrane, integral component of membrane, and vesicle (Figure 4D). For molecular functions (MF), primary enrichment occurred for anion binding, signaling receptor binding, hydrolase activity, and carbohydrate derivative binding (Figure 4E). The KEGG pathways with the highest significance of enrichment for these aging-related crosstalk genes included cytokine–cytokine receptor interaction, chemokine signaling pathway, viral protein interaction with cytokine and cytokine receptor, and phagosome (Figure 4F).

### Construction of PPI networks

**Figure 5:**
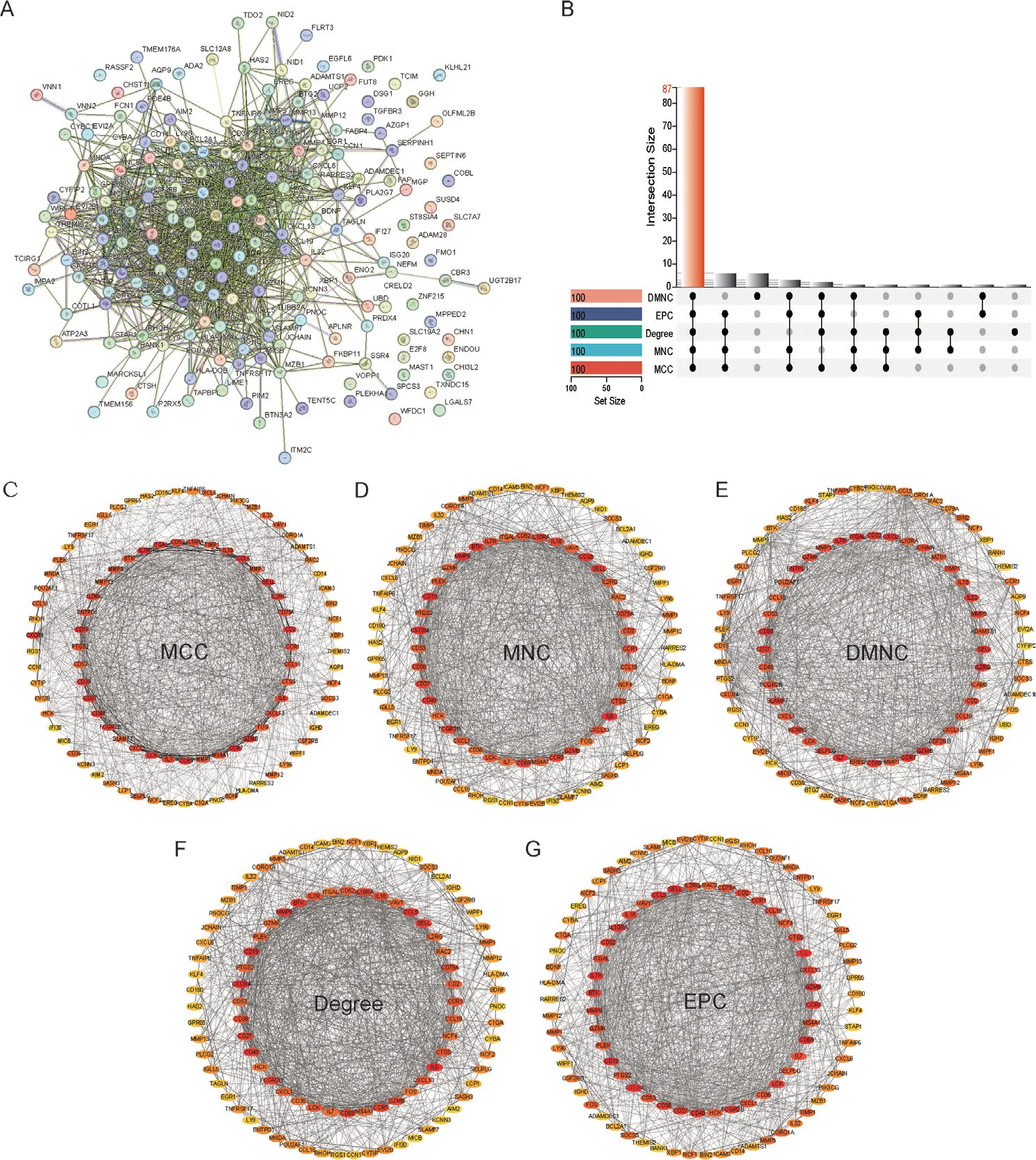
Construction of protein–protein interaction (PPI) networks. (A) The PPI network of aging-related crosstalk genes. (B) The UpSet graph of the intersection of five algorithms as implemented by the plug-in CytoHubba. (C) Aging-related crosstalk genes ranked by maximal clique centrality (MCC). (D) Aging-related crosstalk genes ranked by maximum neighborhood component (MNC). (E) Aging-related crosstalk genes ranked by density of maximum neighborhood component (DMNC). (F) Aging-related crosstalk genes ranked by the degree method. (G) Aging-related crosstalk genes ranked by edge percolated component (EPC). The rank of a protein is indicated by color intensity (from yellow to red), i.e. a higher rank of a protein is indicated by a darker red color.

To explore the interactions among these aging-related crosstalk genes, we constructed a PPI network via the STRING database (Figure 5A). The top 100 nodes were scored and visualized using five algorithms implemented in the CytoHubba plugin for Cytoscape (Figure 5C–G). Further screening using the R package ‘UpSet’ resulted in 87 node genes (Figure 5B).

### Candidate biomarkers identified through machine learning

**Figure 6:**
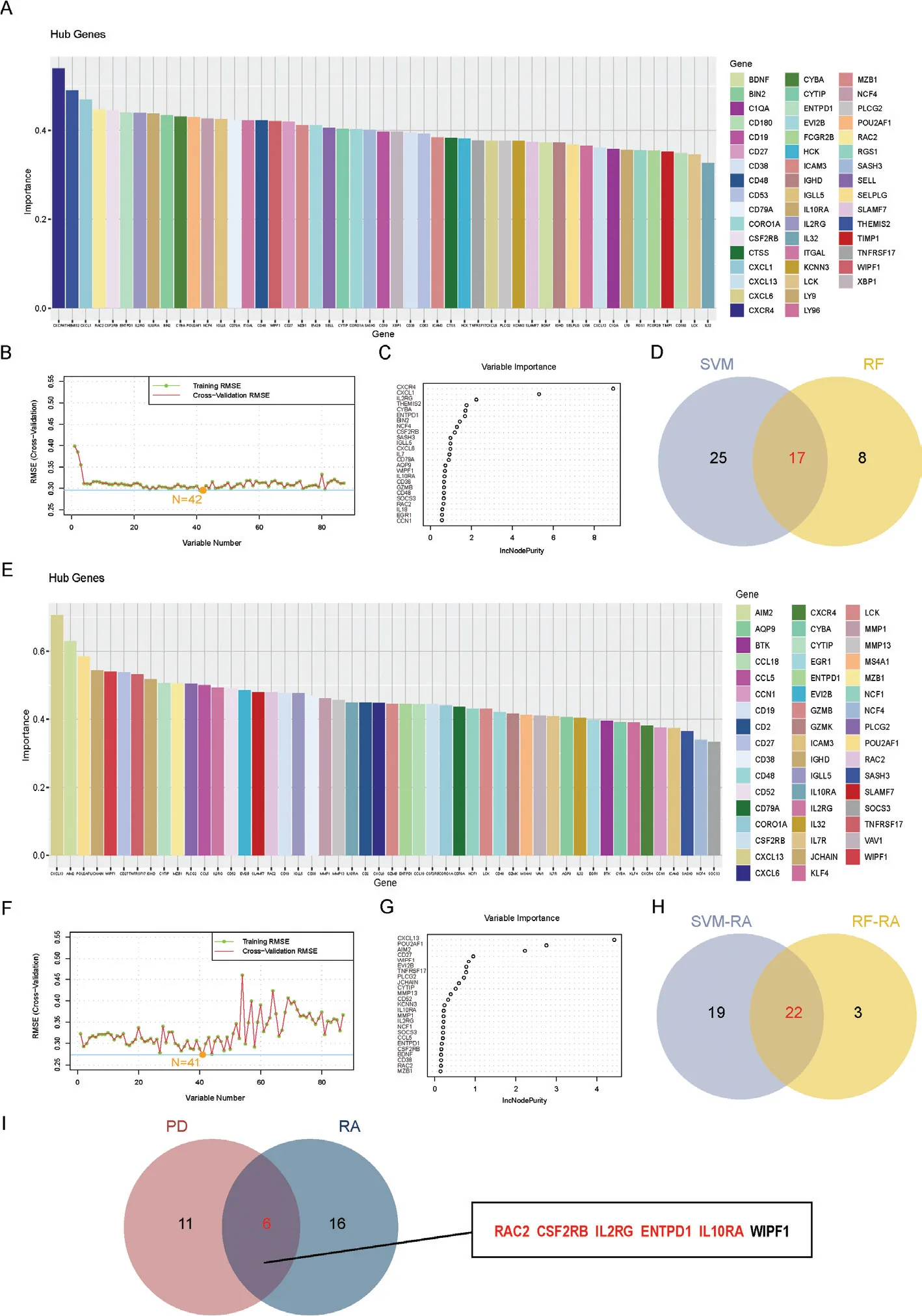
Candidate biomarkers identified through machine learning. (A) Plot of importance scores for genes for PD diagnosis. (B) The top 42 genes were selected based on SVM-RFE with the lowest error and highest accuracy in PD. (C) The top 25 genes were selected and ranked based on the random forest algorithm’s importance score in PD. (D) Venn diagram showing the intersected genes obtained by two machine learning algorithms in PD. (E) Plot of importance scores for genes for RA diagnosis. (F) The top 41 genes were selected based on SVM-RFE with the lowest error and the highest accuracy in RA. (G) The top 25 genes were selected and ranked based on the random forest algorithm’s importance score in RA. (H) Venn diagram showing the intersected genes obtained by two machine learning algorithms in RA. (I) The final hub genes were overlapped by Venn diagram in PD and RA. SVM-RFE: support vector machine-recursive feature elimination.

Based on these 87 node genes, we adopted two machine learning algorithms to filter for potential candidate biomarkers. In the PD dataset, the SVM-RFE algorithm pinpointed 42 genes featuring the minimum root mean squared error (RMSE) (Figure 6A and B), while the RF algorithm selected the top 25 genes according to their importance scores (Figure 6C). The overlap of outcomes derived from the two algorithms resulted in 17 shared genes for the PD group (Figure 6D). Similarly, in the RA dataset, SVM-RFE identified 41 genes (Figure 6E and F), and RF selected the top 25 genes (Figure 6G), resulting in 22 intersection genes for the RA group (Figure 6H). Venn diagram analysis confirmed Rac family small GTPase 2 (RAC2), CSF2RB, IL2RG, ENTPD1, IL10RA, and WIPF1 as common hub genes (Figure 6I).

### Validation in external datasets

**Figure 7:**
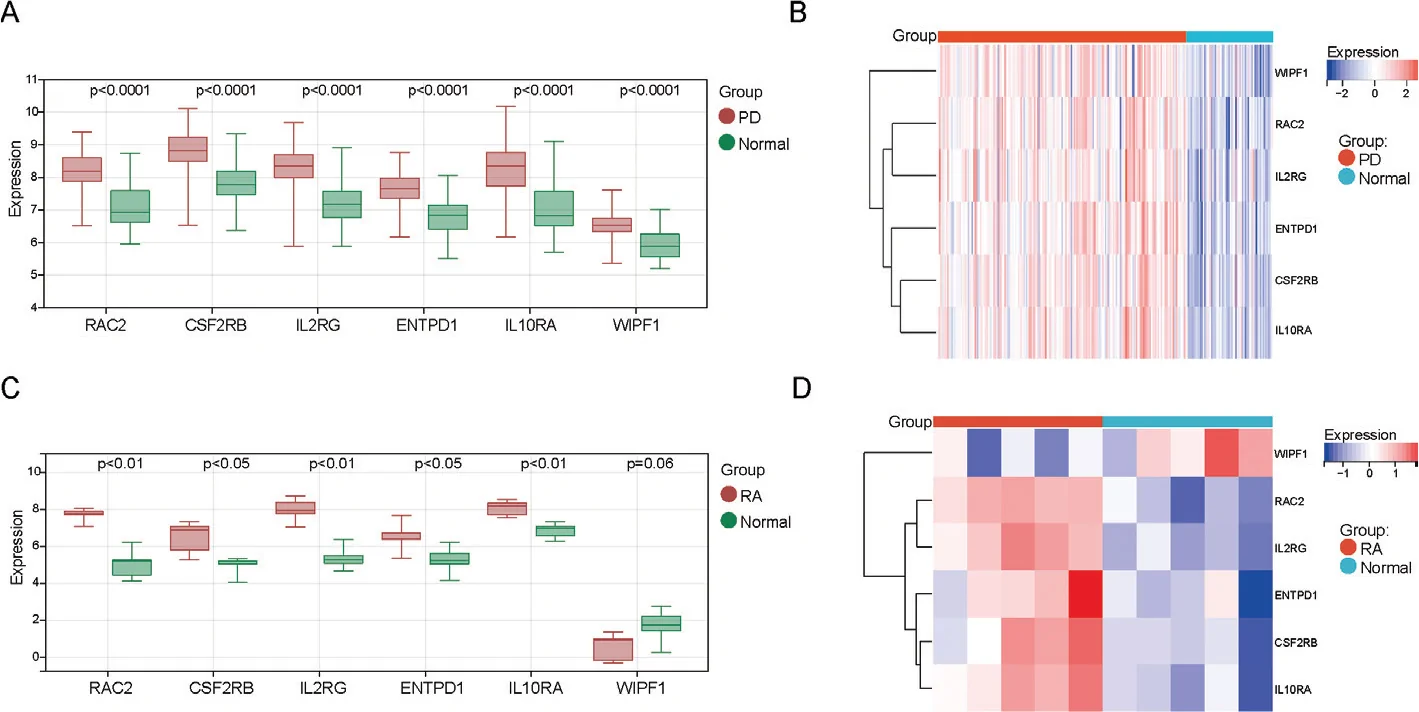
Validation with external datasets. (A) Boxplot of the six hub genes in GSE10334. (B) Heatmap plot of the six hub genes in GSE10334. (C) Boxplot of the six hub genes in GSE1919. (D) Heatmap plot of the six hub genes in GSE1919.

We validated the expression levels of the 6 hub genes in the GSE10334 (PD) and GSE1919 (RA) datasets, with the results visualized through boxplots and heatmaps. In GSE10334, the expression of RAC2, CSF2RB, IL2RG, ENTPD1, IL10RA, and WIPF1 was notably higher in PD tissues when compared with normal tissues (Figure 7A and B). In GSE1919, the expression of RAC2, CSF2RB, IL2RG, ENTPD1, and IL10RA was notably upregulated in RA tissues relative to normal tissues (Figure 7C and D). It could be inferred that RAC2, CSF2RB, IL2RG, ENTPD1, and IL10RA were capable of acting as candidate biomarkers common to both PD and RA.

### Construction of nomograms and evaluation of diagnostic efficacy for candidate biomarkers

**Figure 8:**
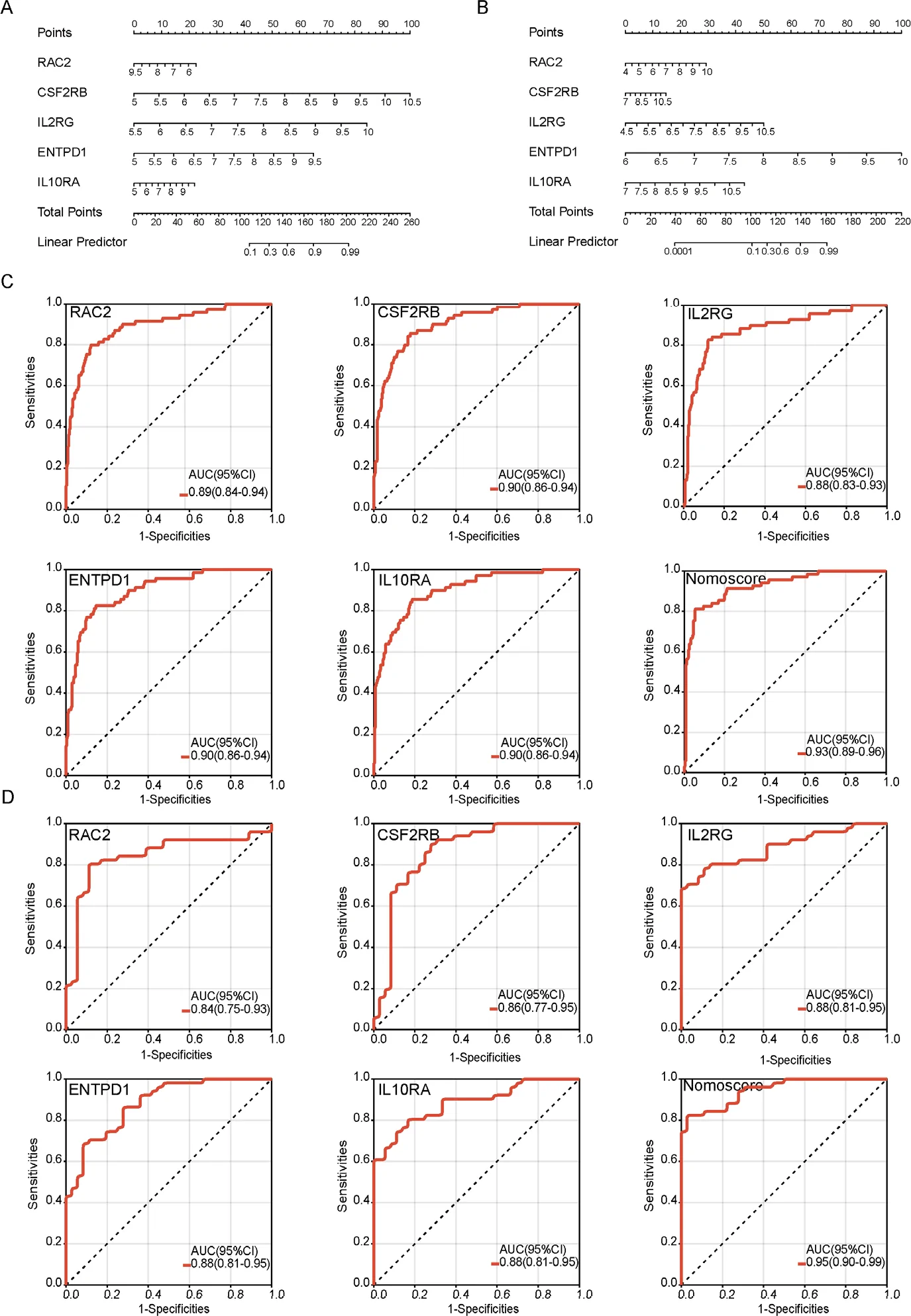
Construction of nomograms and evaluation of diagnostic efficacy for candidate biomarkers. (A, B) Nomogram construction of the five hub genes in datasets for PD and RA. (C) ROC curve of the five hub genes and nomogram in datasets for PD. (D) ROC curve of the five hub genes and nomogram in datasets for RA. ROC: receiver operating characteristic.

Based on the five candidate core genes, we built predictive nomograms for PD and RA (Figure 8A and B). To assess the diagnostic specificity and sensitivity of each candidate gene as well as the constructed nomograms, ROC curves were plotted. We further computed the AUC and corresponding 95% confidence interval (CI) for each analytical item. The findings were presented below: In the PD dataset, RAC2 (AUC 0.89, CI 0.84–0.94), CSF2RB (AUC 0.90, CI 0.86–0.94), IL2RG (AUC 0.88, CI 0.83–0.93), ENTPD1 (AUC 0.90, CI 0.86–0.94), IL10RA (AUC 0.90, CI 0.86–0.94), and nomogram (AUC 0.93, CI 0.89–0.96) (Figure 8C); in the RA dataset, RAC2 (AUC 0.84, CI 0.75–0.93), CSF2RB (AUC 0.86, CI 0.77–0.95), IL2RG (AUC 0.88, CI 0.81–0.95), ENTPD1 (AUC 0.88, CI 0.81–0.95), IL10RA (AUC 0.88, CI 0.81–0.95), and nomogram (AUC 0.95, CI 0.90–0.99) (Figure 8D). All candidate genes demonstrated high diagnostic values for both PD and RA, while the established nomogram displayed the optimal diagnostic performance.

### Immune cell infiltration analysis

**Figure 9:**
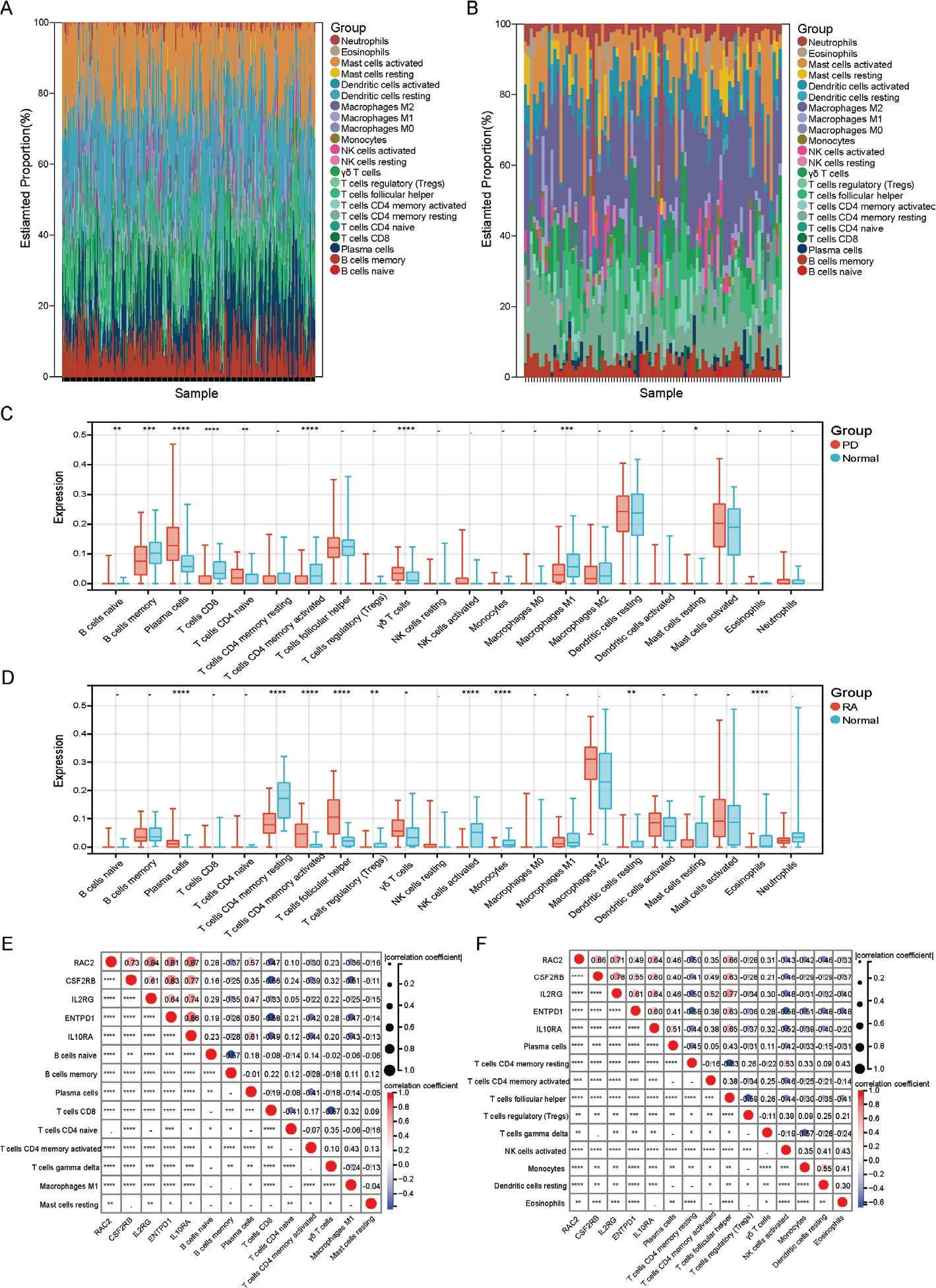
Immune cell infiltration analysis. (A, B) The bar plot visualizing the proportion of infiltrating immune cells in different samples for PD and RA. (C, D) The boxplot comparing the proportion of immune cells between disease and normal for PD and RA. *p < 0.05; **p < 0.01; ***p < 0.001; and ****p < 0.0001. (E, F) Correlation analysis between candidate biomarkers and immune cells in PD and RA.

Drawing on the pathway analysis results presented earlier, the immune system seemed to exert a considerable impact on both PD and RA. To quantitatively characterize immune cell infiltration patterns, we adopted the CIBERSORT algorithm to compute immune cell enrichment scores in PD and RA samples, with the results visualized through bar plots and boxplots. As shown in Figure 9A and 9B, the proportional distributions of 22 immune cell subtypes across individual samples were presented in the bar plots. Compared to normal tissues, PD tissues showed notably increased abundances of naïve B cells, plasma cells, naïve CD4 T cells, γδ T cells, and resting mast cells, while levels of memory B cells, CD8 T cells, activated memory CD4 T cells, and M1 macrophages were decreased in a significant manner (Figure 9C). In the RA group, plasma cells, activated memory CD4 T cells, follicular helper T cells, and γδ T cells were significantly increased, while memory resting CD4 T cells, regulatory T cells (Tregs), activated natural killer (NK) cells, monocytes, resting dendritic cells, and eosinophils were significantly decreased (Figure 9D). Notably, PD and RA samples exhibited a similar trend of increase in plasma cells and γδ T cells.

Correlation analysis (|*r|* > 0.60 and *p* < 0.05) indicated that in the PD group, IL10RA (*r =* 0.61 and *p* < 0.0001) was positively associated with plasma cells (Figure 9E). In the RA group, RAC2 (*r =* 0.66 and *p* < 0.0001), CSF2RB (*r =* 0.63 and *p* < 0.0001), IL2RG (*r =* 0.77 and *p* < 0.0001), ENTPD1 (*r =* 0.63 and *p* < 0.0001), and IL10RA (*r =* 0.65 and *p* < 0.0001) showed a positive association with follicular helper T cells (Figure 9F).

## Discussion

This study adopted a set of comprehensive bioinformatics analytical approaches and machine learning techniques to systematically explore the shared genetic characteristics and molecular mechanisms underlying PD and RA within the aging-related context. We first identified 191 aging-related crosstalk genes, whose functions were significantly enriched in immune-inflammatory pathways. Subsequently, via PPI network analysis, two machine learning methods, and validation with external datasets, five core hub genes (RAC2, CSF2RB, IL2RG, ENTPD1, and IL10RA) were further screened. These genes were significantly highly expressed in both PD and RA tissues, and the diagnostic model constructed based on them demonstrated excellent diagnostic performance for both diseases. Immune infiltration analysis further revealed coordinated dysregulation of plasma cells and γδ T cells in both conditions. These collective findings underscored the central hub of immune-inflammatory dysregulation underlying the comorbidity mechanisms of PD and RA in the context of aging.

Aging serves a pivotal function in the pathogenic mechanisms of chronic inflammatory disorders, such as PD and RA [39, 40]. Undoubtedly, aging facilitates the progression of pathological processes, induces immune system dysregulation, and poses a harmful challenge in coping with chronic inflammatory states, thus accelerating the process of bone loss [41, 42]. It increases the incidence and severity of PD, causes changes in the composition of oral microorganisms and leads to weakened immunity and deterioration of health conditions [43]. Furthermore, the accumulation of autoantibodies is related to physiological changes, and it exhibits a notably positive correlation with the acceleration of biological aging and increased susceptibility to RA [44]. In our research, the proportion of aging-related genes among the crosstalk genes shared by PD and RA was relatively higher, which points to a crucial connection between aging and these two diseases. Concurrently, functional enrichment analysis of the aging-related crosstalk genes in PD and RA confirmed that their biological functions primarily revolve around cytokine–cytokine receptor interaction, chemokine signaling pathway, immune system process, and immune response. This underscores that age-related alterations in immune system function and a chronic low-grade inflammatory state constitute a shared pathophysiological background for PD and RA. With advancing age, immune system dysregulation not only impairs the host’s ability to clear periodontal pathogens but also promotes the aberrant activation and persistence of autoimmune responses in RA [45, 46].

Immune system dysregulation is likely among the most common intrinsic characteristics shared by these two conditions [47]. Changes in innate immunity, along with elevated expression of pro-inflammatory cytokines, infiltration of monocytes, and diminished bacterial clearance, all potentially contribute to the exacerbation of PD [48]. Meanwhile, the excessive production of pro-inflammatory cytokines and RANKL serves as a central driver of RA pathogenic mechanisms. These inflammatory mediators promote joint damage by inducing synovial fibroblasts and chondrocytes to release matrix metalloproteinases (MMPs), as well as by triggering osteoclast differentiation – ultimately resulting in the breakdown of cartilage and bone [49–52]. These similar elements suggest a plausible link between immune dysregulation and both conditions. Recent single-cell RNA sequencing studies provide further support for the immune infiltration pattern identified in the present study. In PD, single-cell analyses of human gingival tissues have revealed inflammation-promoting immune subsets and strengthened macrophage–T/B cell communication within periodontal lesions [53]. Other gingival single-cell atlases have revealed marked changes in plasma cells, B cells, T/NK-lineage cells, mast cells, and myeloid cells associated with PD [54, 55]. These findings are in line with our observation that naïve B cells and plasma cells were increased in PD tissues, while the reduction in memory B cells, CD8 T cells, activated memory CD4 T cells, and M1 macrophages may reflect the redistribution and remodeling of immune-cell states within chronically inflamed gingival lesions. In RA, a large-scale single-cell atlas of synovial tissues classified RA synovium into distinct inflammatory subtypes, including T/B cell-enriched phenotypes characterized by adaptive immune activation and lymphoid-cell accumulation, thereby supporting the increased plasma cells, activated CD4 memory T cells, and T follicular helper cells detected in our analysis [56]. Single-cell immune profiling of seropositive RA synovium further identified activated CD4+ T cells, including T peripheral helper/T follicular helper-like programs that provide B-cell help and participate in local autoimmune responses, providing a basis for the coordinated elevation of T-cell-related inflammatory components in RA [57].

In addition, the immune cell infiltration analysis revealed that analogous changes occurred in plasma cells and γδ T cells in both PD and RA tissues. Among these, plasma cells in PD establish specific communication networks with immune cell subsets via macrophage migration inhibitory factor as a key signaling hub [58]. In RA, plasma cells secrete rheumatoid factor and ACPAs, which attack the synovial membrane and drive chronic inflammation and joint destruction [59]. As major producers of IL-17, γδ T cells fuel tissue damage and inflammatory bone loss [60, 61]. Their hyperactivation may contribute to gingival tissue destruction in PD and chondrocyte damage in RA. These shared immune cell infiltration patterns demonstrate, at the cellular level, common characteristics of immune-inflammatory imbalance underlying both diseases within the context of aging.

Under the shared framework of aging and immune inflammation, the five hub genes we identified (RAC2, CSF2RB, IL2RG, ENTPD1, and IL10RA) may constitute a core molecular network linking aging with both diseases. RAC2 is a highly conserved rat sarcoma (RAS)-related guanosine triphosphate (GTP) hydrolase of the Rho subfamily. Acting as a molecular switch, this enzyme regulates a wide range of downstream biological processes through its cyclic transition between inactive (guanosine diphosphate [GDP]-bound) and active (GTP-bound) states [62]. RAC2 is a key activator of reduced nicotinamide adenine dinucleotide phosphate (NADPH) oxidase [63]. As humans age, intracellular RAC2 undergoes sustained overactivation, prompting NADPH oxidase to generate substantial levels of reactive oxygen species (ROS). This activates the ASK1-JNK/p38 pathway, accelerating the aging or dysfunction of immune cells, thereby weakening the adaptive immune response [64]. RAC2 dysregulation has been implicated in autoinflammatory features and increased susceptibility to infections [65], readily inducing NLRP3 inflammasome activation, resulting in macrophage secretion of IL-18 and IL-1β, which can promote activation and inflammatory responses in fibroblast-like synoviocytes (FLSs) in RA [66] and leukocyte migration in PD [67].

CSF2RB serves as the shared signal-transducing subunit of high-affinity receptors targeting IL-3, IL-5, and colony-stimulating factor (CSF). Through the activation of Janus kinase 2 (JAK2), it facilitates intracellular signaling. This results in phosphorylation at specific tyrosine residues, thereby promoting blood cell production and influencing the proliferation and function of immune cells [68]. Notably, age-related alterations in the hematopoietic system are closely associated with CSF2RB. During aging, hematopoietic stem cells exhibit a bias toward myeloid differentiation [69], a process believed to be linked to the sustained activation of the GM-CSF/IL-3/IL-5 signaling pathway mediated by CSF2RB, thereby laying the foundation for chronic inflammation and immune imbalance [70]. Previous studies have confirmed that CSF2RB overexpression has been linked to autoimmune conditions, including systemic lupus erythematosus (SLE) and multiple sclerosis (MS) [71]. A recent study indicated strong correlations of CSF2RB with Tregs and monocytes in PD and primary Sjögren’s syndrome (pSS) [72].

IL2RG (interleukin-2 receptor gamma chain protein) is a common functional subunit for multiple interleukin receptors and is essential for lymphocyte development and function [73]. Aging is typically accompanied by profound remodeling of the adaptive immune system, with impaired T-cell function representing a core feature [74]. Age-related thymic involution may further contribute to this process by reducing naïve T-cell output and narrowing the T-cell receptor repertoire, thereby favoring peripheral T-cell homeostatic imbalance [75]. As a crucial component of the common gamma chain, altered signaling intensity of IL2RG is considered one of the key factors contributing to diminished T-cell responsiveness and immune dysfunction during aging [76]. Age-related immunosenescence and inflamm-aging not only disrupt T-cell homeostasis but also lead to elevated levels of pro-inflammatory cytokines and impaired tissue repair capacity [77]. In this process, aberrant expression or functional impairment of key immune regulatory genes like IL2RG may further compromise immune homeostasis, predisposing aging individuals to higher comorbidity susceptibility for both diseases.

ENTPD1, alternatively referred to as CD39, belongs to the ectonucleoside triphosphate diphosphohydrolase family and localizes to the surface of innate and adaptive immune cell subsets [78], playing a vital role in immunomodulation. Aging leads to increased cellular damage and death in the organism, resulting in sustained, low-level release of extracellular ATP (eATP) [79]. This forces persistent activation of the CD39/CD73 pathway, causing long-term elevation of adenosine levels in the tissue microenvironment. Chronic exposure to high adenosine concentrations inhibits the functional activity of multiple immune cell populations, including T lymphocytes and NK cells, thereby impairing their ability to combat infections and eliminate cancerous cells [80]. This directly contributes to immunosenescence and exacerbates tissue functional decline and the onset of age-related diseases. Mediated by the adenosine axis, CD39 plays a role in regulating the expression of MMP-1 in gingival fibroblasts induced by IL-1 [81]. Studies have shown that CD39 is a quantitative trait locus related to RA, which affects the levels of CD39+ and CD4+ regulatory T cells [82]. Low expression of CD39+ Tregs in RA patients correlates with methotrexate resistance, suggesting its potential as a biomarker for predicting treatment response [83, 84].

IL10RA, also known as the alpha subunit of the interleukin-10 receptor, acts as a key receptor in the signal transduction of the anti-inflammatory cytokine IL-10, mediating its immunosuppressive functions and thereby effectively inhibiting the synthesis of pro-inflammatory cytokines [85]. This receptor participates in immune regulation and cell survival control by inducing tyrosine phosphorylation of JAK1 and Tyk2 [86]. Although prior studies have mainly concentrated on the functional role of IL10RA in inflammatory bowel disease [87], emerging evidence indicates its potential importance in both PD and RA. The phenomenon of inflamm-aging during aging can lead to dysregulation of immune homeostasis and compromise anti-inflammatory mechanisms such as the IL-10/IL10RA signaling pathway [88]. This not only exacerbates periodontal tissue destruction and alveolar bone resorption [89] but also promotes the progression of synovitis and joint erosion in RA [90]. Consequently, the diminished responsiveness of IL10RA signaling may represent one potential factor contributing to the increased comorbidity of PD and RA in aging individuals. Existing studies have identified certain correlations between IL10RA levels and periodontal parameters [91], and the IL10RA rs9610 polymorphism may increase susceptibility to RA [92], further supporting the hub role of this gene in connecting these two chronic inflammatory diseases within the context of aging.

Taken together, these comprehensive findings suggest that five hub genes may constitute a bridge connecting aging with immune-inflammatory dysregulation, playing a role in both PD and RA. The enrichment of immune-related pathways in aging-related crosstalk genes, the functional association of hub genes with immunosenescence and inflamm-aging, and the coordinated dysregulation of plasma cells and γδ T cells in both diseases together support a framework that age-related immune dysregulation contributes to the exacerbation of these two conditions. These multiple layers of evidence provide a new perspective for understanding how aging may influence the common pathogenesis of PD and RA.

This study is the first to apply comprehensive bioinformatics analysis to investigate the common gene characteristics and molecular mechanisms between PD and RA within the context of aging. These findings may carry potential clinical implications and lay an important foundation for developing combined intervention strategies and biomarkers. However, our study also has some limitations. First, the analytical findings of this study are highly contingent upon the quality and heterogeneity of the selected public database datasets. Although the PD datasets included only nonsmokers, individual-level smoking information was unavailable for the RA datasets. Other key clinical and genetic information, such as human leukocyte antigen (HLA) type, PTPN22 polymorphism, and autoantibody status, was also incomplete. Meanwhile, all GEO datasets lacked uniform sample-level age information. These limitations prevented multivariable adjustment for relevant confounders, evaluation of potential smoking–age interactions, and direct adjustment for age-dependent gene expression, thereby introducing potential confounding bias. Future prospective studies should systematically collect detailed clinical data, including smoking exposure, genetic background, autoantibody status, and age-matched information, to delineate their independent and interactive effects in PD-RA comorbidity. Second, the set of aging-related genes used in this study was obtained from the GeneCards database according to the median association score threshold. Although this strategy is widely adopted in bioinformatics research, it is inherently an operational definition that integrates multisource evidence and may harbor biases originating from the data sources. Future studies with sufficient resources could employ cross-validation across multiple databases or age-stratified transcriptomic data to provide more rigorous evidence for the aging relevance of the identified genes. Third, because synovial biopsy is not a routine clinical procedure for RA and is costly, the RA synovial tissue dataset used here had a limited sample size. Moreover, detailed clinical annotations, such as ACPA status, were incomplete and inconsistent across the dataset, restricting the assessment of whether RA serological heterogeneity influences the shared aging-related transcriptomic signature. Subsequent investigations should utilize larger, well-annotated synovial tissue cohorts to further validate and refine our findings. Furthermore, our research primarily focused on bioinformatic analyses. The functional roles of the identified biomarkers and their associated signaling pathways will need to be further confirmed through experimental validation.

In conclusion, our study identified five shared core hub genes, common immune-inflammatory pathways, and immune cell infiltration characteristics between PD and RA within the aging context. We also suggested that age-associated immune dysregulation and chronic low-grade inflammation may contribute to the exacerbation of both diseases. These findings provide an important theoretical basis for developing biomarkers and joint intervention strategies targeting PD-RA comorbidity against the backdrop of population aging.

## Supplementary Material



## Data Availability

We analyzed seven publicly accessible datasets (GSE16134, GSE10334, GSE55235, GSE55457, GSE12021, GSE77298, and GSE1919) for this research. All these datasets were retrieved from the GEO database (https://www.ncbi.nlm.nih.gov/geo/).
